# Supplementary material for: Content-rich biological network constructed by mining PubMed abstracts
Source: BMC Bioinformatics. 2004 Oct 8;5:147. doi: 10.1186/1471-2105-5-147 (PMC528731; doi:10.1186/1471-2105-5-147)
Supplement: Additional File 2 — The original results of the above study (non-essential files are deleted to keep the file size under the limit set by BMC bioinformatics). [file 1471-2105-5-147-S2.bz2 › chilibotAdditionalFile2/dip20/649ID10368269E11037/html/HYDA_HYDB.html]

 


 **HYDA** and **HYDB** 
  
Found 12 abstracts in PubMed, retrieved 12.  
 

 What does Google say? 
 PDF only 
| .edu only 

---

**Interactive relationship** (e.g. stimulation, inhibition, etc)

**Non-interactive relationship** (e.g. studied together, co-existance, homology, etc.)

- The  **hydA**  and  **hydB**  genes, which map near minute 59 of the genome map, 17 kb distant from each other, are not structural hydrogenase genes, but mutation in either of these genes leads to failure to synthesize any of the hydrogenase isoenzymes.  Ref: 2200847 J Gen Microbiol, 1990
- Identification of proteins encoded in Escherichia coli  **hydA** ,  **hydB**  and analysis of the  **hydA**  locus.  Ref: 2157623 FEMS Microbiol Lett, 1990
- The hydrogenase Hyd isolated from the cytoplasmic membrane of Wolinella succinogenes consists of three polypeptides  **HydA** ,  **HydB**  and HydC and contains cytochrome b 6.4 mumol g protein, which was reduced upon the addition of H2.  Ref: 1587288 Eur J Biochem, 1992
- This is encoded by three genes, hydC gamma,  **hydB**  beta and  **hydA**  alpha, organized within a single operon that contains five additional open reading frames ORFs.  Ref: 12605255 J Biol Inorg Chem, 2003
- The DNA fragments containing the two genes  **hydA**  and  **hydB**  were cloned into a plasmid vector, pBR322.  Ref: 3884595 J Bacteriol, 1985
- **HydA**  and  **HydB**  also showed similarity to the small and the large subunits of periplasmic Ni hydrogenases.  Ref: 1587288 Eur J Biochem, 1992
- The translocation of  **HydA**  and  **HydB**  as well as the maturation of the precursor protein of  **HydA**  appear to depend on the presence of the twin arginine motif.  Ref: 10525739 Arch Microbiol< MedlineTA>Arch Microbiol, 1992
- DNA fragments from Proteus vulgaris and Chromatium vinosum were isolated which restored hydrogenase activities in both  **hydA**  and  **hydB**  mutant strains of Escherichia coli.  Ref: 2200847 J Gen Microbiol, 1990
- The results suggest that  **HydA**  and  **HydB**  are translocated across the membrane by the Tat twin arginine translocation system.  Ref: 10525739 Arch Microbiol< MedlineTA>Arch Microbiol, 1990
- These results, based on complementation analysis with cloned DNA fragments, show that both  **hydA**  and  **hydB**  genes are essential for the production of active hydrogenase.  Ref: 3884595 J Bacteriol, 1985
- **hydA**  and  **hydB** , the genes encoding the large 46 kDa and small 13.  Ref: 10217791 J Bacteriol, 1999
- The plasmids containing the hyd genes pSE 290 and pSE 111 carrying the  **hydA**  and  **hydB**  genes, respectively were used to genetically map a total of 51 mutant strains with defects in hydrogenase activity.  Ref: 3884595 J Bacteriol, 1985
- Mature  **HydB**  and the precursor protein of  **HydA**  were located exclusively in the cytoplasmic cell fraction of the mutant, which catalyzed the reduction of benzyl viologen by H 2, suggesting that  **HydB**  contained Ni.  Ref: 10525739 Arch Microbiol< MedlineTA>Arch Microbiol, 1985
- The enzyme catalyzed the reduction of dimethyl naphthoquinone with H2, in contrast to an earlier preparation which was made up of  **HydA**  and  **HydB**  only and did not contain cytochrome b Unden, G.  Ref: 1587288 Eur J Biochem, 1992
- The hydrogenase proteins synthesized by  **hydA**  and  **hydB**  mutant strains of E. coli transformed by cloned genes from P. vulgaris and C. vinosum were shown by isoelectric and immunological methods to be E. coli hydrogenase.  Ref: 2200847 J Gen Microbiol, 1990
- The genes encoding the subunits were clustered in a single operon in the order hydC gamma,  **hydB**  beta, and  **hydA**  alpha.  Ref: 10482784 Biochim Biophys Acta, 1999
- The three genes were arranged in the order  **hydA** ,  **hydB** , hydC, with the transcription start site in front of  **hydA** , and were present only once on the genome.  Ref: 1587288 Eur J Biochem, 1992
- The amino acid sequences derived from  **hydA** ,  **hydB**  and hydC were similar to those of the membrane Ni hydrogenases of seven other bacteria.  Ref: 1587288 Eur J Biochem, 1992
- The pairs of  **hydA**  and  **hydB**  genes are highly homologous.  Ref: 2661538 J Bacteriol, 1989
- The class II Hyd mutants can be further subdivided into two groups  **hydA**  and  **hydB**  based on the cotransduction characteristics with cys and srl.  Ref: 3884594 J Bacteriol, 1985
